# Fibromyalgia-Like Syndrome Associated with Parkinson’s Disease—A Cohort Study

**DOI:** 10.3390/jcm8081118

**Published:** 2019-07-28

**Authors:** Ran Abuhasira, Yair Zlotnik, Anat Horev, Gal Ifergane

**Affiliations:** 1 Clinical Research Center, Soroka University Medical Center, Beer Sheva, Israel; 2 Faculty of Health Sciences, Ben Gurion University, Beer Sheva, Israel; 3 Department of Neurology, Soroka University Medical Center, Beer Sheva, Israel

**Keywords:** Parkinson’s disease, central pain, cohort studies, fibromyalgia, epidemiology

## Abstract

Parkinson’s disease (PD) and fibromyalgia (FM) are two relatively common disorders that are considered distinct diagnoses. The aim of this study was to investigate the epidemiological characteristics of patients with both PD and FM, as well as their comorbidities and medication use. We performed a population-based retrospective cohort study in Israel from 2000 to 2015. We identified patients with PD according to a refined medication tracer algorithm and patients with FM according to their medical records. Using the algorithm, we identified 2606 patients diagnosed with PD, 60 of them (2.3%) were also diagnosed with FM. Most of the patients were females (88.3%) and the mean age of FM diagnosis was 63.95 ± 12.27 years. These patients had a higher prevalence of depression, anxiety, and dementia. Of the patients diagnosed with PD + FM, 46 (76.7%) were diagnosed with FM after the diagnosis of PD. Patients with PD + FM used analgesics of distinct kinds in higher rates, as well as more anti-PD medications. We suggest that patients with PD + FM represent a distinct subgroup with a fibromyalgia-like syndrome associated with Parkinson’s disease (FLISPAD). Their PD is more treatment resistant, and they take more medications, both analgesics and anti-PD.

## 1. Introduction

Parkinson’s disease (PD) is a common neurodegenerative disease caused by dopaminergic neuron loss [1]. PD is characterized by motor symptoms that mainly include bradykinesia in combination with resting tremor or rigidity [2]. PD also has a distinct prodromal stage identified by non-motor symptoms, such as olfactory dysfunction, constipation, urinary dysfunction, depression, anxiety, and pain [3,4,5,6]. Chronic pain is a common and often underreported symptom of PD, with a reported prevalence of up to 85% in some studies [7,8,9].

Fibromyalgia (FM) is a common disorder characterized by widespread musculoskeletal pain and is often accompanied by other symptoms such as fatigue, sleep disturbances, memory problems, and mood difficulties [10]. Laboratory and imaging tests are often used only for exclusion of other diagnoses and reveal no specific pattern in FM patients [11]. FM is associated with various comorbidities, usually divided into two groups: functional and organic. These functional diseases are termed central sensitivity syndromes (CSS) and include, in addition to FM, chronic fatigue syndrome, irritable bowel syndrome (IBS), temporomandibular disorder, restless legs syndrome, and others. The organic diseases associated with FM include rheumatoid arthritis, systemic lupus erythematosus (SLE), hypothyroidism, inflammatory bowel diseases (IBDs), and others [12].

The value of recognizing FM associated with an organic disease is high, as it helps the physician to consider issuing FM treatment instead of declaring failure in the treatment of the organic disease. The association between FM and PD has been suggested due to shared clinical and possible pathophysiological features, some of which are: muscle and joint stiffness, unusual pelvic and rectal discomfort, poor sleep, fatigue and depression [13,14,15]. Notwithstanding these overlapping symptoms, to date only one case-report has been published addressing a patient with PD + FM [16].

Since PD and FM are two relatively common disorders, it is not uncommon for a neurologist, rheumatologist, or a pain specialist to encounter a patient suffering from both illnesses. Hence, there is a need to characterize this unique group of patients. The aim of this study was to investigate the epidemiological characteristics of patients with PD + FM, as well as their comorbidities and medication use.

## 2. Materials and Methods

### 2.1. Study Design

We performed a population-based retrospective cohort study of all the members of the largest health maintenance organization (HMO) living in southern Israel from January 1, 2000 to December 31, 2015. This HMO (Clalit Health Services—CHS) insures approximately 70% of the population of south Israel. The remaining 30% are insured by one of three other HMOs. The population is similar between all HMOs, except for a higher mean age of CHS members [17]. By law, all Israeli citizens are insured by an HMO of their choice. The HMO insurance includes subsidization of a large and updated list of medications, including almost all anti-parkinsonian drugs (APDs). Soroka University Medical Center (SUMC) is a public tertiary medical center and is the only hospital for the population of south Israel.

### 2.2. Data Sources

The CHS database includes demographic, clinical, laboratory, and medication prescription data of all CHS members. Data on medication purchases include brand and generic name, date, and dosage of purchase. Since all APDs are substantially subsidized by the government, we can assume nearly complete capture with this medication purchasing information. Data on comorbidities were based on the International Classification of Diseases (ICD-9), obtained from community clinics and from hospitalization records. Data from community clinics include also diagnoses not listed in the ICD-9. Prescribed medications were coded according to the World Health Organization’s (WHO) Anatomical Therapeutic Chemical (ATC) classification system.

### 2.3. Parkinson’s Disease and Fibromyalgia Definitions

We identified patients with Parkinson’s Disease (PD) according to a refined medication tracer algorithm. As previously described by Chillag-Talmor et al. [18], this algorithm has demonstrated 96% sensitivity with few false positives—only 4% of patients with non-PD related dyskinesia/spasticity syndromes received false PD identification.

We identified HMO members in the Negev region of southern Israel who purchased 1+ APD within 15 years. These medications include (ATC group codes N04): dopa and dopa derivatives, adamantane derivatives, dopamine agonists, monoamine oxidase (MAO) B inhibitors, and other dopaminergic agents. Pramipexole, which was not available during the creation of the algorithm, was categorized in group 2 of the study drug groups. Patients under 20 years or 85+ years old at the time of their first APD purchase were excluded. The algorithm defines PD at three levels of certainty: definite, probable, and possible. The aim is to differentiate between patients with PD to those with parkinsonism, gait disorders, essential tremor, and non-PD related dyskinesia/spasticity. The algorithm is based on follow-up time, medication purchase intensity, age at first medication purchase and the medication combination purchased. For example, definite PD was defined if the records showed high purchase intensity (9 purchase months out of 12) of either levodopa or dopamine agonists for over 24 months in patients with a follow-up period of over 3 years [18]. For this study, we included only patients with definite PD, according to the algorithm.

Fibromyalgia diagnosis was identified by records from community clinics. These records include all the diagnoses given to patients, both by specialists such as rheumatologists and by family physicians. The accuracy of the diagnosis by family physicians was validated in a previous study in this sample with an overall sensitivity and specificity of FM diagnoses by family physicians of 87.4% and 88.3%, respectively [19]. These high accuracy rates allow us to be convinced that patients with fibromyalgia diagnosis probably meet the criteria for the disease and suffer from the characteristic symptoms, regardless of the physician who diagnosed them.

### 2.4. Statistical Analysis

PD diagnosis date was defined as the first APD purchase for each patient identified with definite PD. Similarly, FM diagnosis date was defined as the first record of the diagnosis during the study period. Use of medications other than APDs was defined as at least one purchase of a specific medication after the first APD purchase. Comorbid conditions included those diagnosed before and after the PD diagnosis. Opioids included ATC group codes N02A; selective serotonin reuptake inhibitors (SSRIs) included ATC group codes N06AB; non-steroidal anti-inflammatory drugs (NSAIDs) included ATC groups codes M01AB and M01AE; neuropathic pain drugs included ATC codes N06AA09, N03AX16, and N03AX12; anxiolytic drugs included ATC groups codes N05B; serotonin–norepinephrine reuptake inhibitors (SNRIs) included ATC codes N06AX16, N06AX17, and N06AX21; anilides (including paracetamol and its combinations) included ATC groups codes N02BE; dipyrone was defined as ATC code N02BB02; pramipeoxle was defined as ATC code N04BC05.

Negative binomial multivariate regression was used to model the number of APDs patients used, adjusted to the total time of their disease. The goal was to identify variables associated with an increased use of APDs for the same period of disease. The dependent variable was the number of different APDs each patient used based on generic names of the drugs (irrespective of dosage levels). Each new generic drug used by the patient, including different generic drugs from the same drug group, was counted as one. Based on the existing clinical knowledge, the following variables were identified a priori: age, sex, and FM diagnosis. Additional variables included: ethnicity, diagnosis of diabetes mellitus or depression. The model was used to estimate the incidence rate ratios (IRRs) of fibromyalgia diagnosis, adjusting for other variables, with the total duration of APD purchase in months as an offset. The purpose of the offset is to adjust for the duration of the PD disease. Parameters were estimated using Fisher method with deviance as a scaling parameter method. When the variance is larger than the mean, the data are said to be over-dispersed. Negative binomial regression was more appropriate than Poisson regression due to over-dispersion found in our data.

A *p*-value of 0.05 or less (two-sided) was considered statistically significant. Statistical analyses were performed using SPSS version 25.0.

### 2.5. Ethics approval and consent to participate

This study was approved by the SUMC institutional review board (IRB) Committee (approval number 0153-15 on 5 May 2016). All clinical investigations were conducted according to the principles expressed in the Declaration of Helsinki. The IRB approval exempted the study from informed consent due to the retrospective data collection nature maintaining subject confidentiality. Patient records were anonymized and de-identified prior to analyses.

## 3. Results

We identified 3838 patients purchasing APDs over the 16-year study period, 1005 (26.2%) of them with possible PD, 227 (5.9%) with probable PD and 2606 (67.9%) with definite PD. Only those with definite PD were further analyzed. Of the patients with definite PD, 60 (2.3%, 95% CI: 1.8%–3.0%) were also diagnosed with fibromyalgia. The mean age of PD diagnosis was 67.9 years; 46.8% of the patients were female. Cardiovascular comorbidities were common among PD patients (Table 1). Variables associated with diagnosis of both FM and PD include younger age, female sex, smoking, not married, diagnosis of depression, anxiety or dementia, without hypertension or heart failure.

**Figure 1:**
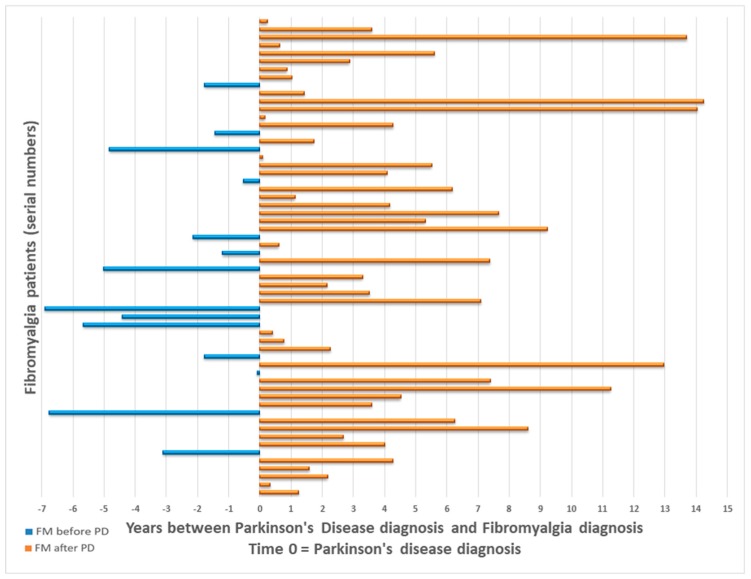
Parkinson’s Disease and Fibromyalgia diagnoses times. Abbreviations: FM—fibromyalgia; PD—Parkinson’s disease.

Mean age of fibromyalgia diagnosis was 63.95 ± 12.27 years (range: 28.5 to 90.1 years). Of those diagnosed with PD + FM, 46 (76.7%) were diagnosed with FM after the diagnosis of PD, with a mean time of 4.5 ± 3.9 years between diagnoses. The 14 remaining PD patients (23.3%) were diagnosed with FM 3.3 ± 2.3 years on average prior to their PD diagnosis (Figure 1).

There were significant differences in medication use between patients with PD only and those with PD + FM. PD + FM patients used distinct analgesics in higher rates, as well as more antidepressants (SSRIs, SNRIs). Of the patients with PD + FM who used antidepressants, most of them (38, 82.2%) purchased the drugs before the diagnosis of FM (mean time 3.6 ± 5.6 years), and 73.1% of the patients with a diagnosis of depression were diagnosed before FM diagnosis. Of the patients with PD + FM who used anxiolytics, most of them (14, 82.3%) purchased the drugs before the diagnosis of FM (mean time 5.2 ± 5.2 years), and 57.1% of the patients with a diagnosis of anxiety were diagnosed before FM diagnosis. The number of APDs from different groups, defined by the medication algorithm, did not differ between patients with PD + FM to those with PD only (Table 2). Median number of admissions to the hospital after PD diagnosis was 2 in the PD only group and 2.5 in the PD + FM group but did not statistically differ (*p*-value = 0.86); the range for the entire cohort admissions number was 0–83 with an IQR of 0–5.

Number of different APDs per patient is reported in Table 3. Every patient used between 1 to 13 different APDs. The result showed that there is an increase of 21.3% in the number of different APDs purchased by patients with FM compared to patients without FM, adjusted to all other variables. This effect of FM diagnosis achieved borderline statistical significance (*p*-value = 0.057). There is also a sex difference in APD purchase—males are expected to purchase 13.6% more APDs compared to females.

## 4. Discussion

As a result of this long-term cohort population-based study, we identified a distinct group of patients with fibromyalgia-like syndrome associated with Parkinson’s disease (FLISPAD). Most of these patients are younger women with a mental health condition (i.e., depression and anxiety). To our knowledge, until now only one case-report has examined concomitant diagnoses of PD + FM [16].

The prevalence of fibromyalgia in the general population ranges from 0.4% to 9.3% [20]. In Israel the range is 2.0–2.6% [21], in accord with our PD sample. Similar to the general population [22,23], the majority of our FM patients were women. The prevalence of smoking in our sample is similar to the Israeli population in the years 2000–2015 [24]. Epidemiologic studies found that cigarette smoking is inversely associated with the risk of developing PD [25], and it has been postulated that the ease of smoking cessation is an early prodromal marker linked to a reduction in nicotine rewarding mechanisms [26]. Cigarette smoking is known to be more prevalent in FM patients, as it is in our cohort; it is possible that nicotine helps patients cope with the disease symptoms [27,28].

Previous studies [20,29], including one conducted in southern Israel [19], showed that FM diagnosis is most common in the fifth decade of life. In our cohort, however, patients received their FM diagnosis at a much older age. Moreover, most FM diagnoses were given a few years after PD diagnosis, or a relatively brief time before the PD diagnosis, in the prodromal stage of disease. This fact may reflect a distinct central pain syndrome, part of the non-motor myriad of PD symptoms. The mean age of fibromyalgia diagnosis in our study was similar to the age in the PD + FM case-report [16].

PD has a well-known prodromal stage characterized by different motor and non-motor symptoms [3], including depression, anxiety [4], and pain [5,6]. FM patients also have elevated prevalence of anxiety and depression [30] and depression is part of the somatic symptoms to consider in the diagnosis of FM according to the American College of Rheumatology 2010 criteria [31]. Furthermore, there are evidence for the association between FM and reduced dopamine metabolism [15]; dopaminergic neuron loss is the central pathology of PD. Thus, the association between FM and PD has been drawn both by epidemiologic data and by plausible neuro-mechanisms [32]. We contend that our group of patients represent a distinct subgroup suffering from fibromyalgia-like syndrome associated with Parkinson’s disease (FLISPAD). They appear to be diagnosed with FM at a somewhat older age, either after the diagnosis of PD or during the prodromal stage of the disease.

This FLISPAD subgroup of patients are mostly female, younger at PD diagnosis with a higher rate of cigarette smoking, anxiety, and depression. As we further showed, these patients consume more analgesic drugs, both over-the-counter (OTC) and prescription medications, including opioids. Rates of opioid consumption in this group was also higher than in previous FM research [33,34]. It should be noted that OTC drugs, paracetamol, dipyrone, and some of the NSAIDs, might be purchased without identification and thus, our data likely underestimates analgesic use. Patients with FLISPAD also use more SSRIs and SNRIs than patients without FM commensurate with the higher depression and anxiety among these patients, as well as probable use of SNRIs to treat chronic pain.

In order to assess treatment adherence, we quantified the number of different APDs patients used during the duration of their disease. Our results, which were close to statistical significance, showed that FLISPAD patients tend to use more APDs than patients without FM (no differences in number of APD groups). This means that the switches were mainly of medications within the same APD group. Other significant predictors of increased APD use were sex, age, and diabetes mellitus. Only some of those factors were also found to be significant to PD treatment adherence in previous studies [35,36].

This may reflect another challenge for physicians treating patients with FLISPAD. The reasons for this greater use of different APDs remain to be elucidated. We believe that there are two possible explanations: (1) based on our personal clinical experience, patients with FM tend to develop more unspecific adverse events when exposed to a new drug, leading the physician to try and exchange between the different APDs available until the patient is content with the drug; (2) recurrent complaints of pain lead physicians to prescribe more dopaminergic drugs, presuming the pain is an expression of dystonic pain or a non-motor manifestation of the OFF state, and hoping that dopaminergic treatment would alleviate this symptom.

FM may coincide with some autoimmune disorders and rheumatic diseases, such as osteoarthritis, rheumatoid arthritis, and systemic lupus erythematosus with rates up to 15–30%, which is significantly higher in these patients than in the general population. This phenomenon was previously termed “secondary FM” but is now better viewed as centralized pain [10,37]. In our cohort, FM prevalence was similar to the general population, but we believe that the late onset of the disease and the coincidence with PD make these patients a specific population.

Pain in PD is common, manifesting in various parts of the body and affecting 30–50% of the patients; distinct types of pain have been described [38]. As a result, there is no consensus on the classification of pain in PD. Classification generally follows the etiology of the pain, and in this regard, Ford’s classification is most commonly used [39]. This classification distinguishes between the following categories: musculoskeletal pain, radicular/neuropathic pain, dystonia-related pain, akathitic discomfort/pain, and central parkinsonian pain. We propose that FLISPAD represents a severe chronic pain syndrome, which may be categorized as a combination of central and musculoskeletal pain. It is interesting to note that the prevalence of FM in PD patients was not higher than in the general population, despite a high prevalence of pain in PD patients. This might be explained by the various types of pain in PD which lead the physicians to attribute most cases of pain in PD patients to PD, as the diagnosis of FM requires that there will not be a disorder that would otherwise explain the pain. However, more research is needed to further elucidate this finding.

The strengths of our study include a prolonged period of data collection with a large cohort of PD patients. The size of the cohort is adequate to detect small effect sizes. Our databases provided us with complete and accurate medication purchase information. Because of the universal drug benefit in Israel, this pharmacy database represents almost all medication purchases in the population, enabling long follow-up, minimal selection bias, and increases the findings generalizability [40,41]. The algorithm used in this study, first described by Chillag-Talmor et al., demonstrated 96% sensitivity and a reasonable rate of false identification of other movement disorders as PD [18].

Our study has several limitations, however. For instance, diagnoses of FM and PD were determined on the basis of patients records and not independently confirmed by a neurologist or a rheumatologist. Yet, as previously noted, FM diagnosis by family physicians in our sample was previously validated [19]. Also, of note, hospital records include ICD-9 codes which do not contain a specific FM code, and that might underestimate FM diagnosis in our cohort. Thus, our understanding is based only upon information from pharmacy information and medical records. The retrospective nature of our study limits our ability to create strict boundaries between pain as a non-motor symptom of PD and pain as part of FLISPAD.

## 5. Conclusions

In summary, we identified the largest cohort to date of PD patients also diagnosed with fibromyalgia. These patients were diagnosed with FM at an older age than other FM patients, and diagnosed with FM after their PD diagnosis or in the prodromal stage of the disease. We contend that this may be a secondary fibromyalgia-like syndrome—or fibromyalgia-like syndrome associated with Parkinson’s disease (FLISPAD). These patients present a challenge for physicians as they use more analgesics, psychotropic medications, and tend to also use more APDs over time. More research is needed to determine the etiology and determinants of this syndrome, the needs of patients and course of treatment, both for PD and FM symptoms.

## Figures and Tables

**Table 1:** Baseline characteristics of Parkinson’s disease patients by diagnosis of fibromyalgia.

Variable	All (*N* = 2606)	No FM (*n* = 2546)	FM (*n* = 60)	*p*-Value (FM vs. No FM)
Age at first APD purchase (years)				
Mean ± SD	67.9 ± 13.7	68.0 ± 13.7	61.3 ± 12.9	**<0.001**
Median (IQR)	71.9 (63.4–77.2)	72.1 (63.8–77.3)	62.9 (55.3–70.0)	
Female (*n*, %)	1220 (46.8%)	1167 (45.8%)	53 (88.3%)	**<0.001**
Comorbidities (*n*, %)				
Coronary artery disease	841 (32.3%)	825 (32.4%)	16 (26.7%)	0.35
Heart failure	342 (13.1%)	340 (13.4%)	2 (3.3%)	**0.02**
Hypertension	1747 (67.0%)	1717 (67.4%)	30 (50.0%)	**0.005**
Diabetes mellitus	1060 (40.7%)	1037 (40.7%)	23 (38.3%)	0.71
Dyslipidemia	1554 (59.6%)	1520 (59.7%)	34 (56.7%)	0.64
COPD	129 (5.0%)	128 (5.0%)	1 (1.7%)	0.37
Depression	559 (21.5%)	533 (20.9%)	26 (43.3%)	**<0.001**
Anxiety	197 (7.6%)	183 (7.2%)	14 (23.3%)	**<0.001**
Dementia	580 (22.3%)	559 (22.0%)	21 (35.0%)	**0.016**
Smoking (*n*, %)	519 (19.9%)	500 (19.6%)	19 (31.7%)	**0.02**
Ethnicity (*n*, %)				
Jewish	2525 (96.9%)	2467 (96.9%)	58 (96.7%)	0.92
Bedouin Arab	81 (3.1%)	79 (3.1%)	2 (3.3%)	
Marital Status (*n*, %)				
Married	1489 (57.1%)	1462 (57.4%)		
Widowed	113 (4.3%)	112 (4.4%)	27 (45.0%)	**0.02**
Divorced	58 (2.2%)	58 (2.3%)	1 (1.7%)	
Single	48 (1.8%)	48 (1.9%)		
First to last APD purchase (years, mean ± SD)	5.8 ± 3.9	5.8 ± 3.9	6.1 ± 4.8	0.63

Abbreviations: FM—fibromyalgia; APD—anti-parkinsonian drug; COPD—chronic obstructive pulmonary disease. Bold indicates statistically significant difference (*p* < 0.05).

**Table 2:** Number of patients who purchased at least one drug of a certain group after first APD purchase.

Variable	All (*N* = 2606)	No FM (*n* = 2546)	FM (*n* = 60)	*p*-Value (FM vs. No FM)
Number of APD groups used by each patient				0.25
Mean	1.79	1.79	1.72	
Median	1	1	1	
Opioids	1387 (53.2%)	1343 (52.7%)	44 (73.3%)	**0.002**
SSRIs	934 (35.8%)	902 (35.4%)	32 (53.3%)	**0.004**
NSAIDs	1566 (60.1%)	1521 (59.7%)	45 (75%)	**0.017**
Neuropathic pain drugs ^a^	433 (16.6%)	402 (15.8%)	31 (51.7%)	**<0.001**
Anxiolytics	911 (35%)	894 (35.1%)	17 (28.3%)	0.28
SNRIs ^b^	288 (11.1%)	264 (10.4%)	24 (40%)	**<0.001**
Anilides (includes Paracetamol and its combinations)	1973 (75.7%)	1926 (75.6%)	47 (78.3%)	0.63
Dipyrone	1528 (58.6%)	1484 (58.3%)	44 (73.3%)	**0.019**

^a^ Includes: pregabalin, gabapentin and amitriptyline. ^b^ Includes: venlafaxine, milnacipran and duloxetine. Abbreviations: FM—fibromyalgia; APD—anti-parkinsonian drug; SSRI—selective serotonin reuptake inhibitor; SNRI—Serotonin–Norepinephrine reuptake inhibitor; NSAIDs—non-steroidal anti-inflammatory drugs. Bold indicates statistically significant difference (*p* < 0.05).

**Table 3:** Negative binomial model for number of different APDs every patient used adjusted to the time of disease

Variable	Incident Rate Ratio	95% CI	*p*-Value
Male	1.136	(1.07 to 1.21)	**<0.001**
Fibromyalgia	1.213	(0.99 to 1.48)	0.057
Diabetes Mellitus	1.145	(1.08 to 1.22)	**<0.001**
Age at first APD purchase	1.017	(1.01 to 1.02)	**<0.001**
Ethnicity	1.164	(0.98 to 1.38)	0.083
Depression	1.067	(0.99 to 1.15)	0.076

Abbreviation: APD—anti-parkinsonian drug. Bold indicates statistically significant difference (*p* < 0.05).

## Data Availability

The data used in the analysis of this study are not publicly available due to the requirements of the IRB Committee, but are available from the corresponding author upon request.
